# Supplementary material for: A Likelihood Approach for Real-Time Calibration of Stochastic Compartmental Epidemic Models
Source: PLoS Comput Biol. 2017 Jan 17;13(1):e1005257. doi: 10.1371/journal.pcbi.1005257 (PMC5240920; doi:10.1371/journal.pcbi.1005257)
Supplement: S1 File — (TAR.GZ) [file pcbi.1005257.s014.tar.gz › HSPH_Online-SI-Revision/IPoi09/n10-severe/n10-severe_BENCH_table4.pdf]

1	R0	{1.68952, 11.8402}	{2.64373, 19.7855}	{{2.64373, 19.7855}, {2.64373, 19.7855}}	{56.4782, 67.1044}	inf	584.	1131.95	{603., 1945.}	94.8766	0.249237	newly	319.	364.286	{182., 652.}	44.3997	0.167983	Reff	0.653675	0.398091	{0.295305, 0.530861}	43.5821	ar	6872.	9088.49	{9032., 9142.}	32.254
2	R0	{1.71739, 9.62661}	{2.64373, 19.7855}	{{2.64373, 19.7855}, {2.64373, 19.7855}}	{53.9388, 105.529}	inf	487.	1795.86	{1214., 2426.}	268.759	0.554279	newly	307.	599.413	{399., 843.}	95.2484	0.273913	Reff	0.669954	0.634663	{0.387306, 1.01255}	39.1141	ar	6874.	9088.49	{9032., 9142.}	32.2155
3	R0	{1.48936, 6.23975}	{2.91171, 17.9732}	{{2.85582, 17.2484}, {2.96797, 18.4042}}	{95.5004, 188.043}	inf	196.	1296.26	{826., 1873.}	561.357	0.794047	newly	176.	446.534	{272., 647.}	153.713	0.374546	Reff	0.69285	0.332269	{0.248338, 0.428373}	53.3884	ar	5666.	9347.85	{9264., 9423.}	64.9815
4	R0	{1.62394, 7.51135}	{2.42205, 19.4475}	{{2.42205, 19.4475}, {2.42205, 19.4475}}	{49.1465, 158.908}	inf	314.	1135.84	{607., 1851.}	261.734	0.516622	newly	227.	380.931	{198., 618.}	56.8839	0.189439	Reff	0.684491	0.542773	{0.346111, 0.725888}	41.8829	ar	6417.	8811.98	{8743., 8883.}	37.3225
5	R0	{1.53632, 8.57652}	{2.42205, 19.4475}	{{2.42205, 19.4475}, {2.42205, 19.4475}}	{57.6527, 126.753}	inf	366.	1135.84	{607., 1851.}	210.538	0.450955	newly	274.	380.931	{198., 618.}	56.2673	0.188562	Reff	0.697643	0.542773	{0.346111, 0.725888}	42.4661	ar	6260.	8811.98	{8743., 8883.}	40.7665
6	R0	{1.47166, 5.7434}	{2.42205, 19.4475}	{{2.42205, 19.4475}, {2.42205, 19.4475}}	{64.5795, 238.606}	inf	206.	1490.29	{834., 2097.}	624.548	0.8296	newly	216.	505.121	{259., 748.}	137.38	0.355882	Reff	0.704484	0.686459	{0.395279, 1.05819}	38.8892	ar	5650.	8811.98	{8743., 8883.}	55.9643
7	R0	{1.48179, 8.87369}	{2.40843, 17.8586}	{{2.36153, 17.2664}, {2.50427, 19.1373}}	{62.5354, 101.253}	inf	206.	974.462	{507., 1613.}	373.04	0.628872	newly	142.	349.369	{180., 535.}	146.988	0.344965	Reff	0.774828	0.507612	{0.331156, 0.745521}	50.975	ar	5233.	8806.68	{8674., 8975.}	68.2912
8	R0	{1.53669, 6.47807}	{2.86656, 18.9813}	{{2.86656, 18.9813}, {2.86656, 18.9813}}	{86.5412, 193.009}	inf	235.	1439.59	{869., 2429.}	512.592	0.758555	newly	203.	481.705	{276., 812.}	137.293	0.340301	Reff	0.682444	0.41333	{0.27691, 0.681095}	50.6701	ar	5934.	9306.52	{9263., 9354.}	56.8339
9	R0	{1.57227, 6.20989}	{2.96947, 15.963}	{{2.96947, 15.963}, {2.96947, 15.963}}	{88.8651, 157.058}	inf	357.	1581.15	{1026., 2423.}	342.898	0.622435	newly	361.	620.629	{375., 977.}	73.4742	0.211602	Reff	0.71271	0.423255	{0.268737, 0.573999}	54.3093	ar	6136.	9383.39	{9320., 9450.}	52.9235
10	R0	{1.54936, 6.50938}	{2.95209, 14.037}	{{2.95209, 14.037}, {2.95209, 14.037}}	{90.5361, 115.643}	inf	238.	949.887	{545., 1560.}	299.112	0.557669	newly	209.	401.677	{222., 656.}	92.7767	0.238402	Reff	0.623308	0.280801	{0.222292, 0.34628}	55.5451	ar	6275.	9365.42	{9314., 9419.}	49.2497
11	R0	{1.42179, 3.18601}	{2.74911, 14.7014}	{{2.59907, 13.5234}, {2.86118, 15.5722}}	{93.3556, 361.435}	inf	53.	1202.22	{569., 2224.}	2168.33	1.3053	newly	90.	502.8	{233., 982.}	458.667	0.693509	Reff	0.684876	0.375601	{0.264325, 0.548774}	49.0765	ar	5322.	9203.31	{9023., 9346.}	72.9295
12	R0	{1.48035, 6.46183}	{2.84728, 16.6676}	{{2.80057, 16.2348}, {2.96398, 17.7348}}	{92.3386, 157.939}	inf	313.	1755.63	{1125., 2316.}	460.903	0.731044	newly	284.	663.125	{407., 888.}	133.495	0.346523	Reff	0.695468	0.513661	{0.313384, 0.608284}	46.6871	ar	5793.	9292.06	{9211., 9391.}	60.4016
13	R0	{1.75755, 8.53854}	{2.85956, 19.8999}	{{2.85956, 19.8999}, {2.85956, 19.8999}}	{62.7015, 133.06}	inf	415.	1417.95	{895., 2112.}	241.675	0.506828	newly	276.	459.836	{280., 684.}	69.1138	0.201871	Reff	0.648009	0.407459	{0.2708, 0.503283}	50.0641	ar	6832.	9302.69	{9253., 9345.}	36.1635
14	R0	{1.47355, 4.89451}	{2.7499, 15.3124}	{{2.7499, 15.3124}, {2.7499, 15.3124}}	{86.6174, 212.848}	inf	140.	1147.32	{598., 1892.}	719.516	0.872171	newly	163.	456.806	{239., 755.}	180.249	0.400891	Reff	0.670023	0.359203	{0.26894, 0.472433}	48.5678	ar	5710.	9203.37	{9154., 9249.}	61.1799
15	R0	{1.62071, 5.98665}	{2.93597, 11.6547}	{{2.93597, 11.6547}, {2.93597, 11.6547}}	{81.1533, 94.6782}	inf	218.	787.361	{471., 1369.}	261.175	0.516248	newly	197.	383.934	{214., 658.}	96.4384	0.251931	Reff	0.578593	0.259506	{0.219611, 0.309158}	55.1488	ar	6635.	9358.84	{9316., 9401.}	41.0525
16	R0	{1.67268, 7.7256}	{2.96947, 15.963}	{{2.96947, 15.963}, {2.96947, 15.963}}	{77.5277, 106.625}	inf	248.	705.29	{357., 1119.}	184.391	0.390102	newly	184.	267.387	{139., 406.}	58.3626	0.160563	Reff	0.552988	0.249373	{0.199548, 0.295462}	55.8896	ar	6947.	9383.39	{9320., 9450.}	35.0711
17	R0	{1.67805, 11.1417}	{2.96271, 18.574}	{{2.90165, 18.0404}, {2.98125, 18.8857}}	{76.5566, 66.7069}	inf	598.	1369.05	{797., 2062.}	128.937	0.333009	newly	353.	459.692	{265., 700.}	42.5496	0.145572	Reff	0.775091	0.368984	{0.244463, 0.446655}	60.2543	ar	6625.	9377.26	{9328., 9437.}	41.5436
18	R0	{1.66743, 9.99046}	{2.85956, 19.8999}	{{2.85956, 19.8999}, {2.85956, 19.8999}}	{71.4951, 99.189}	inf	590.	1683.31	{1173., 2425.}	185.307	0.436429	newly	394.	540.918	{334., 816.}	43.8379	0.146172	Reff	0.682146	0.486256	{0.301112, 0.643687}	47.288	ar	6899.	9302.69	{9253., 9345.}	34.8411
19	R0	{1.70787, 9.85478}	{2.42205, 19.4475}	{{2.42205, 19.4475}, {2.42205, 19.4475}}	{41.817, 97.3408}	inf	473.	1315.74	{688., 1976.}	179.999	0.418741	newly	313.	442.569	{244., 698.}	54.4067	0.183915	Reff	0.632595	0.606394	{0.365003, 0.870243}	38.303	ar	6867.	8811.98	{8743., 8883.}	28.3236
20	R0	{1.53809, 10.5848}	{2.30371, 19.6625}	{{2.30371, 19.6625}, {2.30371, 19.6625}}	{49.7773, 85.7617}	inf	613.	1335.38	{821., 1943.}	123.337	0.349058	newly	373.	441.418	{272., 667.}	35.6861	0.154134	Reff	0.804882	0.634827	{0.430563, 0.845692}	36.1056	ar	6108.	8636.65	{8547., 8714.}	41.3991
21	R0	{1.59925, 6.74479}	{2.96307, 14.3219}	{{2.96307, 14.3219}, {2.96307, 14.3219}}	{85.2787, 112.34}	inf	243.	1066.06	{592., 1912.}	338.71	0.597111	newly	202.	447.242	{235., 819.}	121.71	0.294394	Reff	0.649775	0.295103	{0.22223, 0.395274}	55.8773	ar	6411.	9382.58	{9331., 9427.}	46.3513
22	R0	{1.38779, 4.40977}	{2.52406, 14.814}	{{2.45983, 14.2543}, {2.55942, 15.0551}}	{81.8762, 235.936}	inf	176.	1358.15	{776., 2008.}	671.674	0.861123	newly	221.	578.603	{317., 905.}	161.825	0.385805	Reff	0.751211	0.526333	{0.344622, 0.806086}	42.472	ar	5154.	8941.9	{8824., 9061.}	73.4943
23	R0	{1.53657, 7.50352}	{2.5539, 15.3312}	{{2.51743, 15.191}, {2.60465, 15.5928}}	{66.2076, 104.321}	inf	330.	978.313	{453., 1573.}	196.458	0.419648	newly	276.	397.375	{182., 644.}	60.6318	0.18611	Reff	0.757222	0.406302	{0.299071, 0.49306}	50.2838	ar	5928.	8993.33	{8884., 9088.}	51.7093
24	R0	{1.71131, 6.24526}	{2.93488, 18.8445}	{{2.93488, 18.8445}, {2.93488, 18.8445}}	{71.499, 201.741}	inf	308.	1908.02	{1270., 2469.}	519.486	0.779678	newly	276.	656.361	{416., 879.}	137.812	0.358917	Reff	0.599814	0.557517	{0.304934, 1.13785}	47.6965	ar	6924.	9360.51	{9324., 9401.}	35.1893
25	R0	{1.4412, 3.07729}	{2.86001, 13.5542}	{{2.86001, 13.5542}, {2.86001, 13.5542}}	{98.4464, 340.459}	inf	56.	1340.89	{727., 2443.}	2294.44	1.34258	newly	93.	601.574	{309., 1093.}	546.854	0.764229	Reff	0.624328	0.37919	{0.253111, 0.563422}	49.0453	ar	5810.	9309.77	{9263., 9353.}	60.237
26	R0	{1.4159, 4.93858}	{2.52814, 11.6507}	{{2.53075, 11.6597}, {2.53075, 11.6597}}	{78.5533, 135.912}	inf	215.	830.714	{409., 1347.}	286.379	0.533277	newly	264.	426.746	{207., 695.}	71.0498	0.195394	Reff	0.740657	0.372038	{0.297363, 0.455788}	50.5596	ar	5255.	8958.24	{8899., 9040.}	70.4708
27	R0	{1.57465, 7.05414}	{2.96307, 14.3219}	{{2.96307, 14.3219}, {2.96307, 14.3219}}	{88.1732, 103.028}	inf	430.	1423.27	{839., 2459.}	230.994	0.489546	newly	370.	603.306	{334., 1060.}	66.5083	0.191212	Reff	0.69946	0.368023	{0.245046, 0.547279}	54.3343	ar	6359.	9382.58	{9331., 9427.}	47.5481
28	R0	{1.41422, 4.62402}	{2.86391, 14.6553}	{{2.86193, 14.6465}, {2.86193, 14.6465}}	{102.508, 216.938}	inf	180.	1362.51	{739., 2371.}	652.767	0.841522	newly	219.	573.033	{317., 982.}	161.659	0.374938	Reff	0.681654	0.380092	{0.253281, 0.55779}	51.1782	ar	5485.	9305.7	{9234., 9372.}	69.6573
29	R0	{1.41901, 3.63017}	{2.74396, 10.5379}	{{2.70795, 10.355}, {2.94803, 11.5745}}	{93.3716, 190.287}	inf	109.	612.841	{325., 904.}	462.24	0.699902	newly	157.	325.635	{173., 474.}	108.846	0.268786	Reff	0.594707	0.28147	{0.232884, 0.325225}	52.9467	ar	5979.	9192.68	{9114., 9369.}	53.7495
30	R0	{1.60719, 4.72984}	{2.9884, 12.8326}	{{2.9884, 12.8326}, {2.9884, 12.8326}}	{85.9394, 171.312}	inf	159.	1158.32	{657., 1895.}	628.505	0.826541	newly	176.	529.129	{299., 845.}	200.641	0.436404	Reff	0.595464	0.297659	{0.225624, 0.372653}	52.5035	ar	6479.	9404.84	{9361., 9451.}	45.1588
31	R0	{1.38175, 6.26468}	{2.16056, 9.76972}	{{2.14082, 9.65117}, {2.14082, 9.65117}}	{56.3641, 55.9493}	inf	255.	523.696	{230., 915.}	112.318	0.280464	newly	236.	313.357	{137., 546.}	59.9576	0.201635	Reff	0.70939	0.45021	{0.367579, 0.550833}	39.7849	ar	5397.	8334.61	{8204., 8449.}	54.4304
32	R0	{1.4947, 8.8847}	{2.24606, 16.668}	{{2.24606, 16.668}, {2.24606, 16.668}}	{50.2683, 87.6034}	inf	294.	829.474	{370., 1279.}	183.852	0.409367	newly	208.	315.737	{130., 527.}	66.169	0.210902	Reff	0.787707	0.486382	{0.373295, 0.64417}	43.1456	ar	5407.	8526.33	{8436., 8612.}	57.6906
33	R0	{1.55891, 8.28978}	{2.42205, 19.4475}	{{2.42205, 19.4475}, {2.42205, 19.4475}}	{55.3682, 134.596}	inf	374.	1135.84	{607., 1851.}	203.969	0.441888	newly	292.	380.931	{198., 618.}	51.24	0.181567	Reff	0.720061	0.542773	{0.346111, 0.725888}	43.58	ar	6239.	8811.98	{8743., 8883.}	41.2403
34	R0	{1.60764, 10.4729}	{2.46477, 18.9032}	{{2.46477, 18.9032}, {2.46477, 18.9032}}	{53.316, 80.4963}	inf	407.	1161.77	{589., 1891.}	187.224	0.425176	newly	229.	398.367	{197., 666.}	80.262	0.228393	Reff	0.67971	0.507307	{0.336195, 0.678058}	42.7178	ar	6495.	8883.57	{8780., 8947.}	36.7755
35	R0	{1.48527, 5.74485}	{2.79496, 15.3264}	{{2.7499, 15.0558}, {2.86118, 15.5722}}	{88.1783, 166.785}	inf	235.	1085.97	{533., 1914.}	362.115	0.61956	newly	239.	429.667	{208., 800.}	85.3556	0.231072	Reff	0.703869	0.341624	{0.244917, 0.441084}	53.6214	ar	5811.	9248.2	{9170., 9334.}	59.1499
36	R0	{1.61457, 9.93364}	{2.64373, 19.7855}	{{2.64373, 19.7855}, {2.64373, 19.7855}}	{63.742, 99.1767}	inf	545.	1579.67	{970., 2338.}	189.847	0.441237	newly	332.	517.667	{318., 816.}	58.6871	0.179718	Reff	0.74577	0.533349	{0.347915, 0.805545}	42.384	ar	6338.	9088.49	{9032., 9142.}	43.3968
37	R0	{1.47831, 7.08139}	{2.5618, 15.0409}	{{2.55942, 15.0551}, {2.55942, 15.0551}}	{73.2924, 112.4}	inf	340.	1343.33	{798., 2099.}	295.098	0.56952	newly	278.	565.937	{317., 900.}	105.379	0.28325	Reff	0.759556	0.509804	{0.340403, 0.741976}	46.1693	ar	5530.	9004.13	{8933., 9085.}	62.8233
38	R0	{1.73445, 9.8858}	{2.98125, 18.4749}	{{2.98125, 18.4749}, {2.98125, 18.4749}}	{71.8845, 86.8832}	inf	569.	1781.73	{1120., 2627.}	213.133	0.473421	newly	367.	613.145	{366., 921.}	68.7308	0.203858	Reff	0.688056	0.459516	{0.274573, 0.640671}	48.5918	ar	6842.	9388.66	{9338., 9439.}	37.221
39	R0	{1.66621, 11.4824}	{2.64373, 19.7855}	{{2.64373, 19.7855}, {2.64373, 19.7855}}	{58.6673, 72.3115}	inf	533.	1579.67	{970., 2338.}	196.373	0.450906	newly	292.	517.667	{318., 816.}	77.7941	0.224906	Reff	0.756459	0.533349	{0.347915, 0.805545}	42.7945	ar	6473.	9088.49	{9032., 9142.}	40.4062
40	R0	{1.60694, 9.95741}	{2.64373, 19.7855}	{{2.64373, 19.7855}, {2.64373, 19.7855}}	{64.5195, 98.7013}	inf	510.	1355.78	{761., 2255.}	165.839	0.392708	newly	314.	441.667	{249., 763.}	51.3649	0.16382	Reff	0.690181	0.455683	{0.316719, 0.646392}	42.6457	ar	6625.	9088.49	{9032., 9142.}	37.1848
41	R0	{1.55128, 4.71911}	{2.99458, 12.6606}	{{2.99519, 12.6436}, {2.99519, 12.6436}}	{93.0392, 168.284}	inf	233.	1064.6	{568., 1639.}	356.908	0.620631	newly	259.	494.935	{262., 736.}	93.5982	0.244614	Reff	0.655881	0.299356	{0.219349, 0.341751}	60.0348	ar	6141.	9409.32	{9360., 9461.}	53.2213
42	R0	{1.60063, 6.81118}	{2.42205, 19.4475}	{{2.42205, 19.4475}, {2.42205, 19.4475}}	{51.3185, 185.523}	inf	295.	1490.29	{834., 2097.}	406.996	0.679023	newly	240.	505.121	{259., 748.}	114.677	0.314858	Reff	0.619924	0.686459	{0.395279, 1.05819}	40.2611	ar	6528.	8811.98	{8743., 8883.}	34.9875
43	R0	{1.56986, 5.44652}	{2.8186, 12.0293}	{{2.8186, 12.0293}, {2.8186, 12.0293}}	{79.5447, 120.862}	inf	202.	779.7	{372., 1418.}	285.99	0.53013	newly	196.	372.567	{183., 678.}	94.4898	0.238024	Reff	0.625118	0.283321	{0.228307, 0.363036}	55.0895	ar	6312.	9275.2	{9219., 9322.}	46.9455
44	R0	{1.47523, 8.07785}	{2.59568, 18.2267}	{{2.59755, 18.2464}, {2.59755, 18.2464}}	{75.9508, 125.638}	inf	323.	1398.21	{804., 2101.}	332.882	0.610647	newly	237.	496.	{271., 796.}	110.195	0.29438	Reff	0.760776	0.54296	{0.336642, 0.895895}	49.8892	ar	5692.	9049.61	{8987., 9121.}	58.9883
45	R0	{1.6417, 7.69429}	{2.96398, 17.7348}	{{2.96398, 17.7348}, {2.96398, 17.7348}}	{80.5433, 130.493}	inf	363.	1516.56	{888., 2423.}	317.786	0.59359	newly	273.	534.226	{299., 900.}	95.9352	0.258328	Reff	0.605951	0.39212	{0.254013, 0.581236}	47.8644	ar	6735.	9378.1	{9330., 9427.}	39.2442
46	R0	{1.38865, 5.83473}	{2.29055, 13.7413}	{{2.29055, 13.7413}, {2.29055, 13.7413}}	{64.948, 135.509}	inf	305.	1182.69	{564., 1949.}	287.768	0.55132	newly	319.	544.727	{248., 944.}	78.5352	0.229293	Reff	0.760702	0.574099	{0.380002, 0.872012}	35.9272	ar	5233.	8615.65	{8528., 8694.}	64.6408
47	R0	{1.45289, 6.87903}	{2.36782, 13.2315}	{{2.36011, 13.1698}, {2.36011, 13.1698}}	{62.9733, 92.3452}	inf	301.	1067.03	{551., 1920.}	254.496	0.504951	newly	259.	505.881	{245., 938.}	97.991	0.25232	Reff	0.779475	0.519176	{0.34906, 0.791345}	44.2315	ar	5379.	8741.64	{8668., 8850.}	62.5143
48	R0	{1.69416, 9.4831}	{2.64373, 19.7855}	{{2.64373, 19.7855}, {2.64373, 19.7855}}	{56.0496, 108.64}	inf	573.	1795.86	{1214., 2426.}	213.413	0.483653	newly	377.	599.413	{399., 843.}	60.1322	0.189923	Reff	0.683255	0.634663	{0.387306, 1.01255}	39.2874	ar	6925.	9088.49	{9032., 9142.}	31.2418
49	R0	{1.47843, 7.16286}	{2.66614, 16.7393}	{{2.65407, 16.6623}, {2.67299, 16.6779}}	{80.3359, 133.696}	inf	252.	1378.25	{768., 2229.}	446.923	0.708334	newly	214.	523.899	{271., 895.}	144.812	0.351939	Reff	0.752669	0.4684	{0.312205, 0.64202}	48.9697	ar	5449.	9124.94	{9064., 9188.}	67.4609
50	R0	{1.68843, 5.72427}	{2.93653, 10.872}	{{2.93653, 10.872}, {2.93653, 10.872}}	{73.9207, 89.9281}	inf	170.	520.738	{274., 900.}	206.316	0.430345	newly	156.	267.262	{133., 454.}	76.4292	0.200784	Reff	0.581326	0.228635	{0.195867, 0.275447}	60.6701	ar	6800.	9367.05	{9316., 9430.}	37.7507
